# Prognostic value of high-sensitivity C-reactive protein in patients undergoing percutaneous coronary intervention with different glycemic metabolism status

**DOI:** 10.1186/s12933-023-01932-2

**Published:** 2023-08-24

**Authors:** Le Li, Shangyu Liu, Zhuxin Zhang, Likun Zhou, Zhenhao Zhang, Yulong Xiong, Zhao Hu, Yan Yao

**Affiliations:** 1 Chinese Academy of Medical Sciences, Peking Union Medical College, National Center for Cardiovascular Diseases, Fu Wai Hospital, Beijing, China; 2 Department of Cardiology, The First Hospital of Hebei Medical University, Shijiazhuang, Hebei China

**Keywords:** Coronary artery disease, Percutaneous coronary intervention, High-sensitivity C-reaction protein, Diabetes mellitus, Survival analysis

## Abstract

**Background:**

High-sensitivity C-reaction protein (hsCRP), a biomarker of residual inflammatory risk, has been demonstrated with poor cardiovascular outcomes. We aimed to investigate the prognostic value of hsCRP in patients undergoing percutaneous coronary intervention (PCI) with or without diabetes mellitus (DM).

**Methods:**

In this large-scale, prospective cohort study, we enrolled 8050 consecutive patients who underwent PCI for coronary artery stenosis. All subjects were stratified as high hsCRP (> 3 mg/L) and low hsCRP (≤ 3 mg/L) and were divided into four groups (hsCRP-L/non-DM, hsCRP-H/non-DM, hsCRP-L/DM, hsCRP-H/DM). The primary endpoint of the study was major adverse cardiovascular events (MACEs), including all-cause mortality, myocardial infarction, stroke, and unplanned vessel revascularization, evaluated at a 3 year follow-up.

**Results:**

After 35.7 months (interquartile range: 33.2 to 36.0 months) of median follow-up time, 674 patients suffered from MACEs. We found elevated hsCRP was highly associated with an increased risk of MACEs in both diabetic (hazard ratio [HR] = 1.68, 95% confidence interval CI 1.29–2.19, P < 0.001) and non-diabetic patients (HR = 1.31, 95% CI: 1.05–1.62, P = 0.007) after adjustment for other confounding factors. Kaplan-Meier survival analysis showed the highest incidence of MACEs in hsCRP-H/DM (P < 0.001). In addition, the results of the restricted cubic spline analysis suggested a positive linear relationship between hsCRP and MACEs.

**Conclusion:**

Elevated hsCRP is an independent risk factors of MACEs in patients undergoing PCI irrespective of glycemic metabolism status.

**Supplementary Information:**

The online version contains supplementary material available at 10.1186/s12933-023-01932-2.

## Introduction

Despite vast improvements in medicine and surgery, coronary artery disease (CAD) remains the leading cause of death worldwide and poses a significant economic burden, although its mortality rate has decreased over recent decades [1]. Inflammation is a crucial pathophysiological basis of CAD, strongly linked to plaque initiation, progression, and sudden fibrous cap rupture [2]. Targeted anti-inflammatory treatments, such as colchicine and other agents, have shown significant benefits in improving cardiovascular outcomes in patients with CAD [3–5]. Previous studies have demonstrated that high-sensitivity C-reactive protein (hsCRP), a vital biomarker reflecting systemic inflammatory status, is an effective predictor of major adverse cardiovascular events (MACEs) in CAD patients [6, 7]. Recently, Ridker PM et al. have confirmed that hsCRP, an inflammation biomarker, is a stronger predictor of future MACEs than low-density lipoprotein cholesterol in 31,245 patients with or at high risk of atherosclerotic disease [8]. This highlights the potential of hsCRP in guiding clinical decisions for cardiovascular therapies. However, the measurement of hsCRP may be influenced by comorbidities, which could limit its clinical application and interpretability as a systemic inflammatory biomarker [9].

Over the past three decades, the global incidence of diabetes mellitus (DM) has quadrupled, with around 415 million people living with the disease worldwide [10]. Subclinical chronic inflammation is a common feature in the natural course of DM, and levels of inflammatory biomarkers such as hsCRP, interleukin-1, and interleukin-10, many of which are secreted by adipocytes, are correlated with prevalent and incident DM [11]. As a risk equivalent of CAD, DM is closely associated with greater atherosclerotic plaque burden and increased risk of poor clinical outcomes [12]. However, limited research has been conducted to clarify the relationship between hsCRP and clinical outcomes in CAD patients with different glycemic metabolic statuses after PCI. Therefore, we aimed to investigate the prognostic impact of hsCRP on patients undergoing PCI with or without DM, based on a large prospective cohort.

## Methods

### Study design

This study was a prospective cohort study conducted at a single center. Between January and December 2013, a total of 10,724 patients who underwent PCI at a large tertiary care center (Chinese Academy of Medical Sciences, Fuwai hospital) were screened consecutively. Patients who were under 18 years old, had inadequate data, had excessive inflammatory conditions, which are defined as known infections (such as respiratory infections) or hsCRP > 10 mg/L, or did not receive a drug-eluting stent (DES) were excluded. As a result, a total of 8050 patients who underwent PCI were included in the analysis. Based on their hsCRP levels and glycemic metabolism status, participants were divided into four groups: hsCRP-L/non-DM (n = 4417), hsCRP-H/non-DM (n = 1189), hsCRP-L/DM (n = 1898), and hsCRP-H/DM (n = 546).

The study process was in accordance with the Declaration of Helsinki and was approved by the Institutional Review Board of Fuwai hospital. All subjects provided informed written consent for long-term follow-up before intervention.

### PCI procedure

The PCI procedure was performed by experienced cardiologists in accordance with the current practice guideline of China. Prior to PCI, patients who were not on long-term aspirin and P2Y12 inhibitors received 300 mg of aspirin and a loading dose of P2Y12 inhibitors. Patients scheduled for primary PCI received the same dose of aspirin and either clopidogrel or ticagrelor, with a loading dose of 300 mg or 600 mg depending on bleeding risk. During PCI, 50–100 U/kg of heparin sodium was used based on bleeding risk. Patients with greater than 70% stenosis in main branch vessels and ischemic symptoms were recommended for coronary stent implantation. In addition, the use of coronary intravascular imaging techniques such as intravascular ultrasound and optical coherence tomography were at the discretion of the treating physician. Following PCI, dual antiplatelet therapy including aspirin (100 mg daily) and either ticagrelor (90 mg twice daily) or clopidogrel (75 mg daily) were prescribed for at least 12 months.

### Definitions and clinical endpoints

Elevated hsCRP was defined as >3 mg/L, as recommended by the Centers for Disease Control and Prevention and the American Heart Association [13]. Type 2 diabetes mellitus (DM) is diagnosed based on the current guideline: fasting plasma glucose (PG) ≥ 126 mg/dL (7.0 mmol/L), or 2 h PG ≥ 200 mg/dL (11.1 mmol/L) during oral glucose tolerance test, or A1C ≥ 6.5% (48 mmol/mol), or in a patient with classic symptoms of hyperglycemia or hyperglycemic crisis, a random plasma glucose ≥ 200 mg/dL (11.1 mmol/L), or patients who have already taken insulin or any oral hypoglycemic agents [14]. The primary endpoint of interest was the composite of major adverse cardiovascular events (MACEs), which including all-cause death, acute myocardial infarction (MI), stroke and unplanned vessel revascularization (VR) within 3 years after PCI. Follow-up visits were performed at 1 month, 6 months, 1 year, 2 years and 3 years after discharge via outpatient clinics or over the telephone or examination of medical records.

### Statistical analyses

The Kolmogorov-Smirnov test was used to evaluate the normal distribution of the data. Continuous variables were expressed as means ± standard deviations (SD) or as median and interquartile range (IQR) of 25th to 75th percentiles depending on the distribution of data, and compared using t–test. Categorical variables were presented as counts and percentages, and were compared using the chi-squared test or the Fisher exact test. The risks of MACEs were evaluated by Kaplan-Meier method using the log-rank test. The Cox proportional hazard regression models were used to assess the relationships between outcomes and potential risk factors. All models were adjusted by age, sex, body mass index (BMI), current smoking, hypertension, hyperlipidemia, previous stroke, previous PCI, previous coronary artery bypass graft (CABG) and SYNTAX score. The relationships between hsCRP and indexes of glucose metabolism, hsCRP and the risk of MACEs were illustrated by a linear regression model and a restricted cubic spline, respectively. All statistical analyses were performed by R software version 4.1.0 (R Foundation for Statistical Computing, Vienna, Austria). All tests were two-tailed and a statistical significance was established at a P< 0.05.

## Results

### Baseline characteristics

In this study, a total of 8050 qualified patients with a mean age of 58.4 ± 10.2 years and 76.6% male was enrolled. A flow diagram depicting the screening process is presented in Additional file 1: Figure S1. Baseline characteristics of the patients stratified by occurrence of MACEs during the follow-up period are summarized in Table 1. Patients who experienced MACEs had significantly higher hsCRP values (1.37 [0.73–2.65] vs. 1.56 [0.84–3.17], P = 0.007) and a higher proportion of diabetes mellitus (37.1% vs. 29.7%, P < 0.001) compared to those who did not experience MACEs. Furthermore, patients with MACEs presented with more severe coronary lesions. The clinical presentation (acute/chronic coronary syndrome) and discharge medications (such as antiplatelet drug and statin) were comparable between the two groups.

**Table 1:** Baseline characteristics stratified by the primary endpoint

Variables	Total (n = 8050)	Non-MACEs (n = 7376)	MACEs (n = 674)	*P* value
Age, years	58.4 ± 10.2	58.3 ± 10.1	60.0 ± 10.7	**< 0.001**
Male	6166 (76.6)	5640 (76.5)	526 (81.3)	0.355
Body mass index, kg/m^2^	25.9 ± 3.2	25.9 ± 3.2	25.9 ± 3.3	0.830
Previous history				
DM	2444 (30.4)	2194 (29.7)	250 (37.1)	**< 0.001**
Hypertension	5155 (64.0)	4696 (63.7)	459 (68.1)	**0.022**
Hyperlipidemia	5400 (67.1)	4940 (67.0)	460 (68.2)	0.500
Current** s**moking	4530 (56.3)	4137 (56.1)	393 (58.3)	0.266
Stoke	834 (10.4)	750 (10.2)	84 (12.5)	0.061
PAD	620 (7.7)	550 (7.5)	70 (10.4)	**0.006**
Clinical history				
Previous PCI	1922 (23.9)	1726 (23.4)	196 (29.1)	**0.001**
Previous CABG	339 (4.2)	295 (4.0)	44 (6.5)	**0.002**
Previous MI	1587 (19.7)	1426 (19.3)	161 (23.9)	**0.004**
Clinical presentation				0.683
ACS	4598 (57.1)	4208 (57.0)	390 (57.9)	
CCS	3452 (42.9)	3168 (43.0)	284 (42.1)	
Laboratory				
hsCRP, mg//L	1.39 (0.73–2.69)	1.37 (0.73–2.65)	1.56 (0.84–3.17)	**0.007**
HbA_1c_, %	6.59 ± 1.19	6.58 ± 1.19	6.71 ± 1.23	**0.006**
Creatinine, mmol/L	74.9 ± 15.4	74.8 ± 15.2	76.9 ± 17.1	**< 0.001**
LDL, mmol/L	2.48 ± 0.91	2.48 ± 0.91	2.48 ± 0.88	0.991
LVEF, %	63.3 ± 7.0	63.5 ± 6.9	62.0 ± 7.8	**< 0.001**
Discharge therapy				
Aspirin	7958 (98.9)	7293 (98.9)	665 (98.7)	0.623
Clopidogrel	7930 (98.5)	7263 (98.5)	667 (99.0)	0.312
Beat-blocker	7246 (90.0)	6647 (90.1)	559 (82.9)	0.302
Statins	7734 (96.1)	7087 (96.1)	647 (96.0)	0.911
Insulin	968 (12.0)	868 (11.8)	100 (14.8)	**0.019**
DAPT	7847 (97.5)	7188 (97.5)	659 (97.8)	0.608
PCI-related data				
Multivessel disease	6074 (75.5)	5494 (74.5)	580 (86.1)	**< 0.001**
LM stenosis	510 (6.3)	464 (6.3)	46 (6.8)	0.586
SYNTAX score	10.0 (5.5–16.0)	10.0 (5.0–16.0)	12.0 (7.0–19.0)	**< 0.001**
TIMI 0 before PCI	1288 (16.0)	1148 (15.6)	140 (20.8)	**< 0.001**
Number of stents	2 (1–2)	2 (1–2)	2 (1–2)	0.117
Target group				**< 0.001**
HsCRP-L/non-DM	4417 (54.9)	4105 (55.7)	312 (46.3)	
HsCRP-H/non-DM	1189 (14.8)	1077 (14.6)	112 (16.6)	
HsCRP-L/DM	1898 (23.6)	1717 (23.3)	181 (26.9)	
HsCRP-H/DM	546 (6.8)	477 (6.5)	89 (13.2)	

Bolded *p*-values indicate statistically significant differences

*MACEs* major adverse cardiovascular events, *DM* diabetes mellitus, *PAD* peripheral arterial disease, *PCI* percutaneous coronary intervention, *CABG* coronary artery bypass graft, *MI* myocardial infarction, *ACS* acute coronary syndrome, *CCS* chronic coronary syndrome, *hsCRP* high-sensitivity C-reactive protein, *HbA1c* glycosylated hemoglobin A1c, *LDL* low density lipoprotein, *LVEF* left ventricular ejection fraction, DAPT dual antiplatelet therapy, LM left main.

The study participants were further divided into four groups based on their hsCRP value and glycemic metabolism status. Compared to diabetic patients with high hsCRP, patients in the other three groups were younger, had a lower BMI, and had a lower proportion of comorbidities such as hypertension, hyperlipidemia, previous stroke, and previous PCI. Patients in the hsCRP-H/DM group had more complex and critical coronary lesions compared to patients in the other groups. Additionally, patients with elevated hsCRP levels tended to have worse metabolic conditions, such as higher levels of glycated hemoglobin (HbA1c) or low-density lipoprotein (LDL) cholesterol. Additional file 1: Table S1 showed that hsCRP was positively correlated with HbA1c (correlation coefficient = 0.025, P < 0.001) and LDL cholesterol (correlation coefficient = 0.044, P < 0.001), regardless of whether the patients had diabetes. The baseline characteristics of the four groups were summarized in Table 2.

**Table 2:** Baseline characteristics stratified by the four groups

Variables	HsCRP-L/non-DM (n = 4417)	HsCRP-H/non-DM (n = 1189)	HsCRP-L/DM (n = 1898)	HsCRP-H/DM (n = 546)	*P* value
Age, years	58.0 ± 10.3	58.6 ± 10.7	59.1 ± 9.5	59.3 ± 9.9	< 0.001
Male	3427 (77.6)	906 (76.2)	1446 (76.2)	387 (70.9)	0.005
Body mass index, kg/m^2^	25.6 ± 3.1	26.3 ± 3.3	26.1 ± 3.1	26.9 ± 3.3	< 0.001
Previous history					
Hypertension	2706 (61.3)	751 (63.2)	1290 (67.0)	408 (74.7)	< 0.001
Hyperlipidemia	2838 (64.3)	750 (63.1)	1401 (73.8)	411 (75.3)	< 0.001
Current** s**moking	2484 (56.2)	717 (60.3)	1021 (53.8)	309 (56.6)	0.006
Previous stoke	368 (8.3)	133 (11.2)	234 (12.3)	81 (14.8)	< 0.001
Previous PAD	304 (6.9)	75 (6.3)	186 (9.8)	55 (10.1)	< 0.001
Clinical history					
Previous PCI	1057 (23.9)	213 (17.9)	526 (27.7)	126 (23.1)	< 0.001
Previous CABG	175 (4.0)	44 (3.7)	89 (4.7)	31 (5.7)	0.143
Previous MI	905 (20.5)	192 (16.1)	399 (21.0)	91 (16.7)	0.001
Clinical presentation					< 0.001
ACS	2473 (56.0)	794 (66.8)	1012 (53.3)	319 (58.4)	
CCS	1944 (44.0)	395 (33.2)	886 (46.7)	227 (41.6)	
Laboratory					
hsCRP, mg//L	1.06 (0.60–1.69)	4.66 (3.62–6.63)	1.10 (0.65–1.81)	4.56 (3.60–6.38)	< 0.001
HbA_1c_, %	6.06 ± 0.58	6.18 ± 0.73	7.68 ± 1.36	7.98 ± 1.41	< 0.001
Creatinine, mmol/L	74.7 ± 14.2	76.5 ± 17.0	74.3 ± 15.8	75.8 ± 18.8	< 0.001
LDL, mmol/L	2.47 ± 0.90	2.70 ± 0.94	2.36 ± 0.87	2.56 ± 0.91	< 0.001
LVEF, %	63.7 ± 6.9	62.7 ± 7.3	63.2 ± 7.0	62.8 ± 7.2	< 0.001
Drug therapy					
Aspirin	4367 (98.9)	1170 (98.4)	1884 (99.3)	537 (98.4)	0.103
Clopidogrel	4351 (98.5)	1166 (98.1)	1871 (98.6)	542 (99.3)	0.285
Beat-blocker	3943 (89.3)	1056 (88.9)	1751 (92.3)	496 (90.8)	0.001
Statins	4259 (96.4)	1140 (95.9)	1807 (95.2)	528 (96.7)	0.116
DAPT	4304 (97.4)	1150 (96.7)	1858 (97.9)	535 (98.0)	0.195
PCI-related data					
Multivessel disease	3148 (71.3)	904 (76.0)	1565 (82.5)	457 (83.7)	< 0.001
LM stenosis	270 (6.1)	61 (5.1)	137 (7.2)	42 (7.7)	0.058
SYNTAX score	9.0 (5.0–15.5)	10.0 (6.0–17.0)	10.0 (6.0–17.0)	11.0 (6.0–17.5)	< 0.001
TIMI 0 before PCI	647 (14.6)	231 (19.4)	309 (16.3)	101 (18.5)	< 0.001
Number of stents	1 (1–2)	2 (1–2)	2 (1–2)	2 (1–2)	< 0.001

Abbreviations as in Table 1

### Primary outcome

**Fig. 1:**
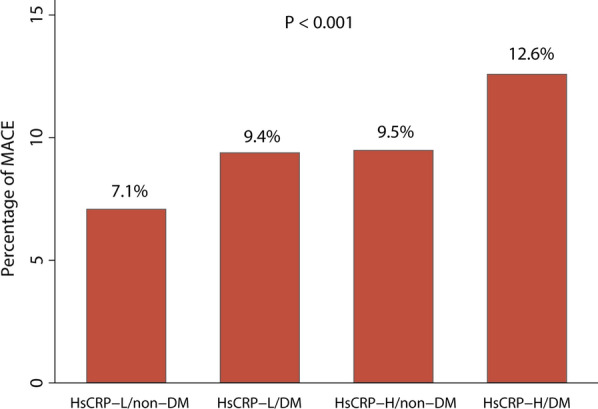
Prevalence of major adverse cardiovascular outcomes in the four groups

**Fig. 2:**
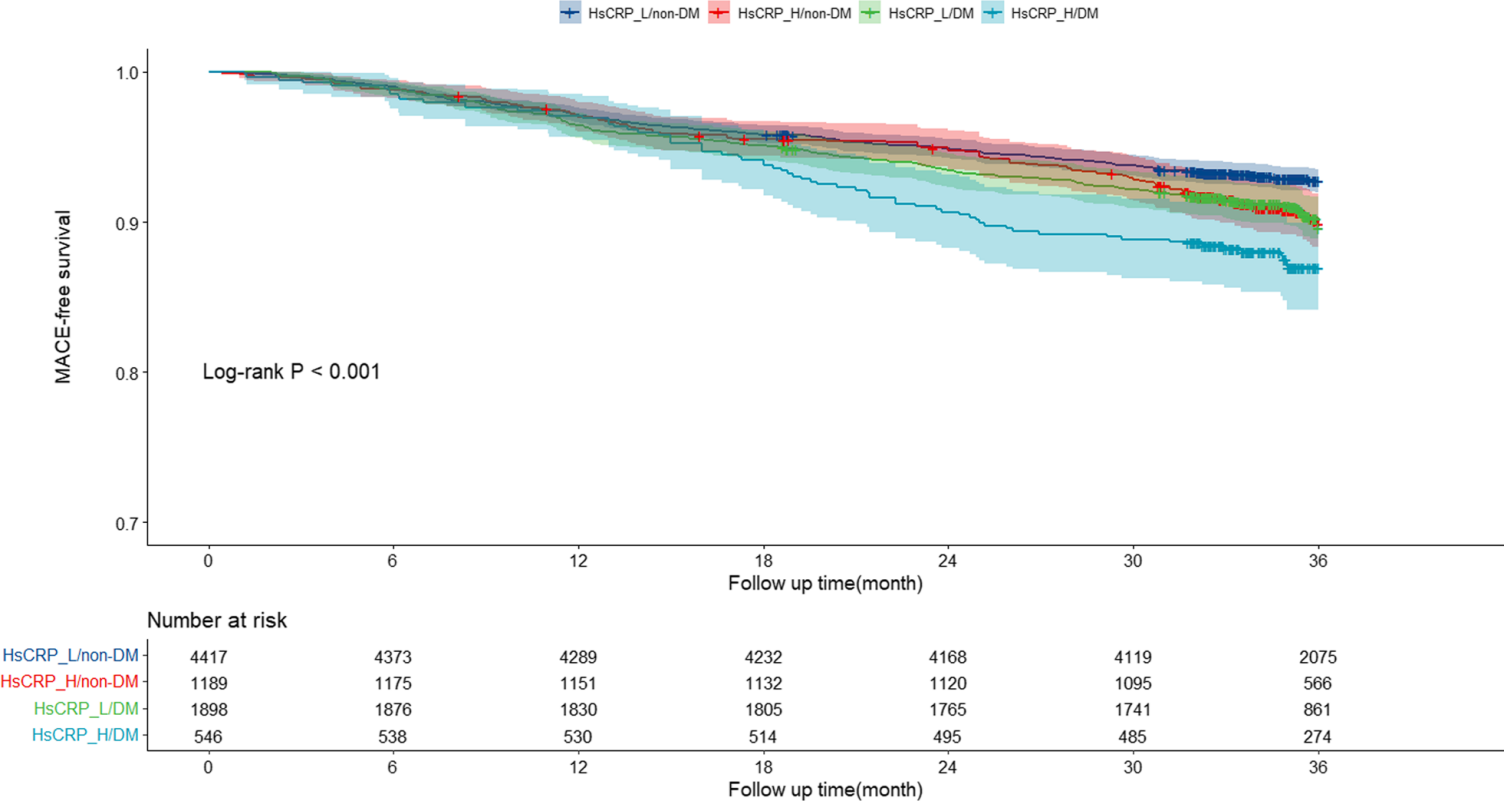
Kaplan–Meier analysis for major adverse cardiovascular outcomes according to different hsCRP levels and glycemic metabolism status

After a median follow-up time of 35.7 months (IQR: 33.2 to 36.0 months), a total of 674 patients experienced MACEs. The percentages of MACEs in the four groups were illustrated in Fig. 1, with the incidence of MACEs in hsCRP-L/non-DM, hsCRP-H/non-DM, hsCRP-H/DM, and hsCRP-H/DM groups being 7.1%, 9.4%, 9.5%, and 12.6%, respectively. Patients with elevated hsCRP and DM had the highest risk of MACEs compared to the other groups (log-rank P < 0.001) (Fig. 2). Furthermore, the Kaplan-Meier analysis curves revealed that both elevated hsCRP and DM were significantly related to an increased risk of MACEs (Additional file 1: Figure S2), and the relationship between elevated hsCRP and MACEs was consistent in both diabetic and non-diabetic patients (Additional file 1: Figure S3). In the adjusted model, the restricted cubic spline (RCS) analysis indicated a linear relationship between hsCRP and the risk of MACEs (P for non-linear association =0.250) (Additional file 1: Figure S4).

Table 3 presents the unadjusted and adjusted hazard ratios (HRs) for patients in the four groups. In the multivariate model, with the hsCRP-L/non-DM group as the reference, the hsCRP-H/non-DM (HR = 1.30, 95% CI 1.05 to 1.62, P = 0.016), hsCRP-L/DM (HR = 1.29, 95% CI 1.07 to 1.55, P = 0.007), and hsCRP-H/DM (HR = 1.68, 95% CI 1.29 to 2.19, P < 0.001) groups were found to be significantly associated with a higher risk of MACEs (see Fig. 4). Furthermore, after adjusting for potential confounders, patients in the hsCRP-H/DM group were identified as an independent risk factor for all-cause mortality, with an adjusted HR of 2.87 (95% CI 1.61–5.09, P < 0.001), whereas this relationship was not observed in the other groups. Additionally, patients in the hsCRP-H/DM group were found to be at the highest risk of MI (HR = 3.05, 95% CI 1.71–5.43, P < 0.001). However, no significant relationships between stroke and either DM or elevated hsCRP were observed in this study. In order to investigate the potential synergistic association between hsCRP and DM in predicting outcomes, we conducted an interaction analysis in this study. However, our findings did not reveal any significant interactions between hsCRP and DM in predicting outcomes (all P for interaction > 0.05).

**Table 3:** Univariate and multivariate Cox regression analyses for MACEs

	Univariate logistic analysis		Multivariate logistic analysis	
	HR (95% CI)	P value	HR (95% CI)	P value
MACEs				
HsCRP-L/non-DM	Ref.	Ref.	Ref.	Ref.
HsCRP-H/non-DM	1.34 (1.04–1.53)	0.008	1.31 (1.05–1.62)	0.016
HsCRP-L/DM	1.36 (1.13–1.64)	0.001	1.29 (1.07–1.55)	0.007
HsCRP-H/DM	1.81 (1.40–2.35)	< 0.001	1.68 (1.29–2.19)	< 0.001
All-cause mortality				
HsCRP-L/non-DM	Ref.	Ref.	Ref.	Ref.
HsCRP-H/non-DM	1.12 (0.60–2.09)	0.713	1.09 (0.58–2.04)	0.779
HsCRP-L/DM	1.63 (1.03–2.61)	0.039	1.49 (0.93–2.39)	0.079
HsCRP-H/DM	3.19 (1.82–5.59)	< 0.001	2.87 (1.61–5.09)	< 0.001
MI				
HsCRP-L/non-DM	Ref.	Ref.	Ref.	Ref.
HsCRP-H/non-DM	1.55 (0.88–2.73)	0.119	1.56 (0.88–2.76)	0.126
HsCRP-L/DM	1.95 (1.24–3.07)	0.004	1.74 (1.10–2.75)	0.018
HsCRP-H/DM	3.43 (1.95–6.03)	< 0.001	3.05 (1.71–5.43)	< 0.001
Stroke				
HsCRP-L/non-DM	Ref.	Ref.	Ref.	Ref.
HsCRP-H/non-DM	0.71 (0.39–1.29)	0.267	0.49 (0.24–1.02)	0.055
HsCRP-L/DM	1.14 (0.73–1.79)	0.554	1.15 (0.71–1.88)	0.565
HsCRP-H/DM	1.92 (0.81–4.58)	0.141	2.20 (0.84–5.73)	0.108
Unplanned VR				
HsCRP-L/non-DM	Ref.	Ref.	Ref.	Ref.
HsCRP-H/non-DM	1.19 (0.83–1.69)	0.346	1.28 (0.89–1.84)	0.180
HsCRP-L/DM	1.60 (1.16–2.20)	0.004	1.53 (1.09–2.12)	0.013
HsCRP-H/DM	1.53 (0.95–2.46)	0.083	1.50 (0.91–2.46)	0.112

### Subgroup analyses

**Fig. 3:**
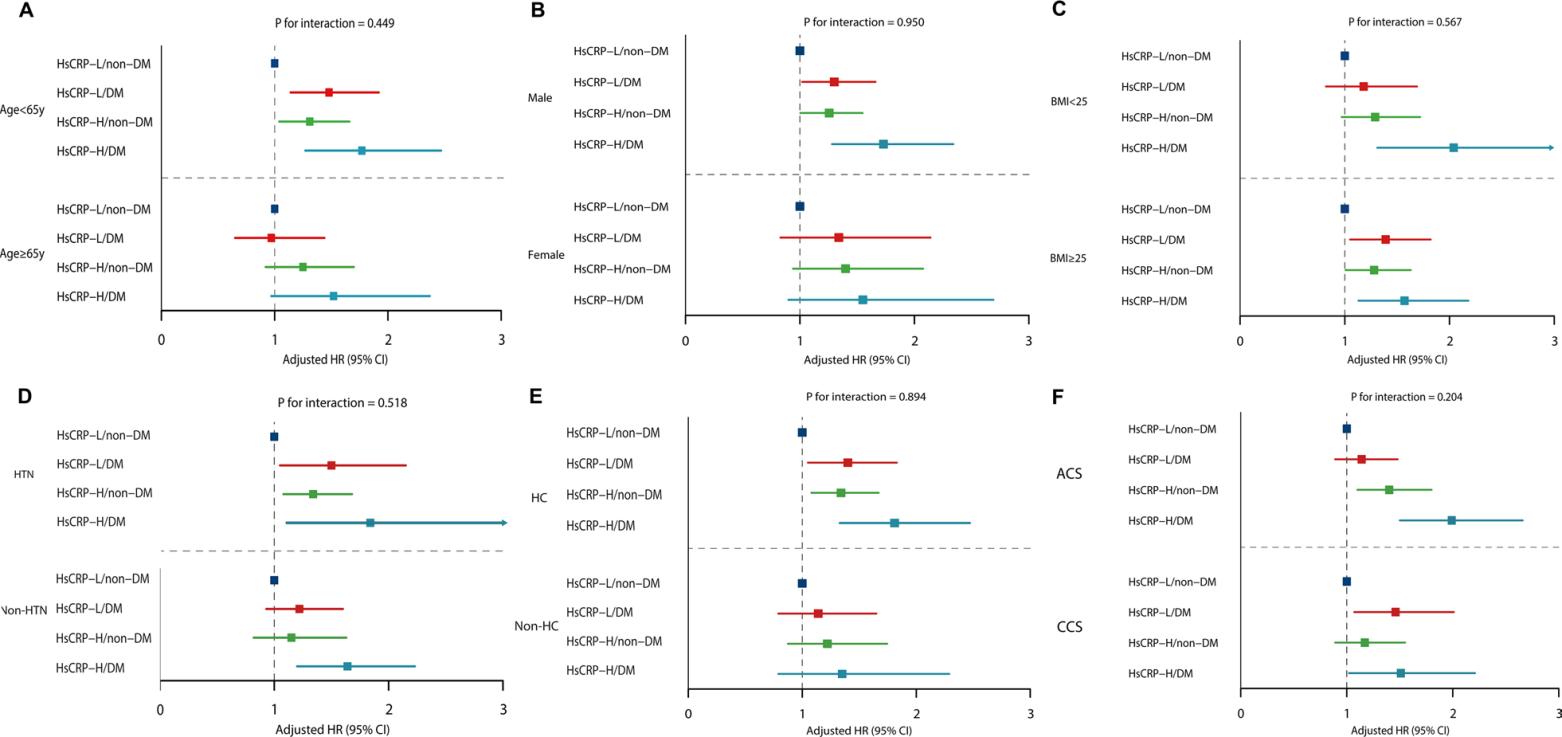
Subgroup analyses. Adjusted by age, sex, body mass index, current smoking, hypertension, hyperlipidemia, previous stroke, previous percutaneous coronary intervention, previous coronary artery bypass graft and SYNTAX score

The potential effects of confounding factors on the associations between MACEs and hsCRP in different glycemic metabolism statuses were further investigated by performing subgroup analyses according to covariates such as age, sex, BMI, hypertension, hypercholesterolemia, and clinical presentation. The results showed that the associations between the four risk groups and MACEs were largely consistent across different subgroups, and hsCRP-H/DM was associated with the highest risk of MACEs in all subgroups. No significant interactions were observed in any of these subgroups, as evidenced by Fig. 3, Additional file 1: Table S2, Table 3.

**Fig. 4:**
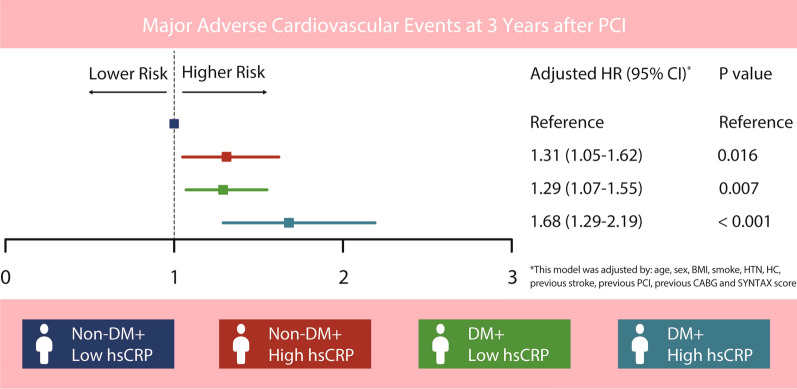
Adjusted hazard ratio for major adverse cardiovascular events according to hsCRP and diabetes mellitus

## Discussion

In summary, this study highlights the important role of hsCRP as a predictor of MACEs in patients undergoing PCI. The results demonstrate that patients with both elevated hsCRP and DM are at the highest risk for MACEs. After adjusting for potential confounders, hsCRP-H/DM is an independent risk factor of all-cause mortality and MI. The findings suggest that hsCRP can serve as a useful biomarker to identify high-risk individuals for MACEs in patients undergoing PCI, and may help to guide clinical management and treatment strategies.

Chronic low-grade inflammation is a key driver of atherosclerosis, which is the underlying pathology of CAD [15]. Our results are consistent with previous studies that have demonstrated a positive association between hsCRP and cardiovascular events in various populations, including patients with angina, acute coronary syndrome, chronic kidney disease, and diabetes [16–19]. The mechanisms underlying this association are not fully understood, but hsCRP has been shown to be a marker of systemic inflammation and oxidative stress, which are known to contribute to the pathogenesis of atherosclerosis [20, 21]. In addition, hsCRP may directly promote the formation of atherosclerotic plaques by activating endothelial cells and promoting the migration and proliferation of smooth muscle cells [22]. In our study, we also found that patients with elevated hsCRP and DM had the highest risk of MACEs. This finding is consistent with previous studies that have shown that patients with both inflammation and DM have a higher risk of cardiovascular events than those with only one of these risk factors [23, 24]. The mechanisms underlying this synergistic effect are not fully understood, but may involve the promotion of endothelial dysfunction, oxidative stress, and inflammation by hyperglycemia and insulin resistance [25–27].

In addition to hsCRP, other biomarkers such as troponin, natriuretic peptides, and myeloperoxidase have also been shown to be associated with cardiovascular events in various populations [23, 28]. However, hsCRP is unique in that it is a relatively stable biomarker that can be measured easily and inexpensively in routine clinical practice. As such, hsCRP may have an important role in risk stratification and clinical decision-making for patients with cardiovascular disease.

The alteration of cardiovascular risk factors is crucial for secondary prevention after PCI [29]. Previous studies have consistently shown that patients with elevated hsCRP are at a higher risk of MACEs, even when they reach target levels of LDL [30]. This finding has been attributed to residual inflammation, as demonstrated by the CANTOS trial [3]. Inflammatory state has also been found to predict adverse outcomes after stent implantation, indicating that anti-inflammatory therapies may have a role in the secondary prevention of patients who have undergone PCI [6]. Our study further highlights that stented patients with both elevated hsCRP and DM are particularly high-risk individuals who may benefit from anti-inflammatory treatments in addition to lipid-lowering therapy.

Our study has important clinical and research implications. Previous studies have shown that both inflammation and hyperlipidemia are strongly associated with future cardiovascular events [31]. Thus, for patients with CAD or a high risk of atherosclerosis, statin therapy is commonly prescribed, and some patients may also receive PCSK9 inhibition treatment. However, previous studies and our findings suggest that even after intensive lipid-lowering therapy, residual inflammatory risk, as measured by hsCRP level, still persists and is significantly associated with poor outcomes [8, 32]. Therefore, for patients who continue to have high residual inflammatory risk despite intensive lipid-lowering therapy, anti-inflammatory treatments should be considered as an adjunct to reduce the risk of future cardiovascular events [33]. In our study, despite more than 95% of participants receiving statin therapy, approximately 21.6% of patients had residual inflammation as evaluated by hsCRP. Elevated hsCRP was found to be highly associated with a higher risk of MACEs in patients undergoing PCI, regardless of their glycemic metabolism status. Our findings suggest that not only residual cholesterol risk but also residual inflammation level should be dynamically evaluated in patients undergoing PCI. Therefore, in the current setting where intensive lipid-lowering therapy is widely accepted, future research involving atherosclerosis should pay more attention to anti-inflammatory agents for reducing residual inflammation risk. For instance, new antidiabetic drugs such as sodium-glucose cotransporter-2 inhibitors and glucagon-like peptide-1 receptor analogues have demonstrated certain cardiovascular benefits, including the ability to lower hsCRP concentrations. This suggests that these agents could improve cardiovascular outcomes through multiple mechanisms, including the aspect of anti-inflammatory activity [34, 35].

## Strength and limitations

The present study has several strengths. First, it is a large-scale, prospective cohort study that included a substantial number of patients with or without DM. Second, we used rigorous statistical methods to adjust for potential confounding variables, which increased the internal validity of our findings. Third, we used data from a nationally representative sample, which enhances the external validity of our results. However, our study has several limitations that should be noted. First, this was an observational study, and as such, we cannot establish causality between hsCRP and MACEs. Second, we did not measure other biomarkers of inflammation or oxidative stress, which may have confounded the relationship between hsCRP and MACEs. Third, we did not quantitively assess the impact of interventions such as statins or lifestyle modifications on hsCRP levels and MACEs.

## Conclusion

In conclusion, our study highlights the utility of hsCRP as a biomarker for identifying high-risk patients for MACEs in patients undergoing PCI with or without DM. It can be a valuable tool for guiding clinical management and treatment strategies. Our findings suggest that patients with both elevated hsCRP and DM are at particularly high risk for cardiovascular events and may require more aggressive risk factor modification and therapeutic interventions, including anti-inflammatory therapies. Further studies should explore the impact of hsCRP on clinical outcomes in other populations and settings, and evaluate its utility as a biomarker for monitoring response to therapy. These efforts will ultimately aid in improving patient care and reducing the burden of cardiovascular disease.

## Supplementary Information

**Additional file 1**: **Table S1. **Relationship between hsCRP and indexes of metabolism by linear regression. **Table S2.** Subgroup analysis for the primary endpoint as the unadjusted model. **Table S3. **Subgroup analysis for the primary endpoint as the adjusted model. **Table S4. **Interaction analysis between hsCRP and DM in predicting outcomes. **Figure S1.** Flow chart. **Figure S2.** Kaplan–Meier analysis for major adverse cardiovascular outcomes according to **A** hsCRP and **B** diabetes mellitus. **Figure S3.** Kaplan–Meier analysis for major adverse cardiovascular outcomes according to different hsCRP level in the DM **A** and non-DM **B** group. **Figure S4.** Restricted cubic splines of hsCRP levels in relation to adjusted HR for the risk of MACEs.

## Data Availability

The datasets used during the current study are available from the corresponding author on reasonable request.
