# Correction: Prognostic value of high-sensitivity C-reactive protein in patients undergoing percutaneous coronary intervention with different glycemic metabolism status

**DOI:** 10.1186/s12933-024-02529-z

**Published:** 2024-12-06

**Authors:** Le Li, Shangyu Liu, Zhuxin Zhang, Likun Zhou, Zhenhao Zhang, Yulong Xiong, Zhao Hu, Yan Yao

**Affiliations:** 1 Chinese Academy of Medical Sciences, Peking Union Medical College, National Center for Cardiovascular Diseases, Fu Wai Hospital, Beijing, China; 2 Department of Cardiology, The First Hospital of Hebei Medical University, Shijiazhuang, Hebei China

**Correction to: Cardiovascular Diabetology (2023) 22:223** 10.1186/s12933-023-01932-2

In the original publication of this article [1], the author noticed the error in the funding number.

The incorrect and correct funding information are presented in this correction article.

Incorrect Funding:

This study was supported by Medical and Health Technology Innovation Project of Chinese Academy of Medical Sciences (2021-CXGC09-1).

Correct Funding:

This study was supported by Chinese Academy of Medical Sciences Innovation Fund for Medical Sciences (CIFMS, 2021-I2M-1-063).
