# Supplementary material for: Prognostic value of high-sensitivity C-reactive protein in patients undergoing percutaneous coronary intervention with different glycemic metabolism status
Source: Cardiovasc Diabetol. 2023 Aug 24;22:223. doi: 10.1186/s12933-023-01932-2 (PMC10463538; doi:10.1186/s12933-023-01932-2)
Supplement: Supplementary file 1 — Additional file 1: Table S1. Relationship between hsCRP and indexes of metabolism by linear regression. Table S2. Subgroup analysis for the primary endpoint as the unadjusted model. Table S3. Subgroup analysis for the primary endpoint as the adjusted model. Table S4. Interaction analysis between hsCRP and DM in predicting outcomes. Figure S1. Flow chart. Figure S2. Kaplan–Meier analysis for major adverse cardiovascular outcomes according to A hsCRP and B diabetes mellitus. Figure S3. Kaplan–Meier analysis for major adverse cardiovascular outcomes according to different hsCRP level in the DM A and non-DM B group. Figure S4. Restricted cubic splines of hsCRP levels in relation to adjusted HR for the risk of MACEs. [file 12933_2023_1932_MOESM1_ESM.docx]

**Prognostic Value of High-Sensitivity C-Reactive Protein in Patients undergoing Percutaneous Coronary Intervention with Different Glycemic Metabolism Status**

**Additional file Online Content**

**Table S1.** Relationship between hsCRP and indexes of metabolism by linear regression **Table S2.** Subgroup analysis for the primary endpoint as the unadjusted model

**Table S3.** Subgroup analysis for the primary endpoint as the adjusted model

**Table S4.** Interaction analysis between hsCRP and DM in predicting outcomes

**Figure S1**. Flow chart.

**Figure S2**. Kaplan–Meier analysis for major adverse cardiovascular outcomes according to (**A**) hsCRP and (**B**) diabetes mellitus.

**Figure S3**. Kaplan–Meier analysis for major adverse cardiovascular outcomes according to different hsCRP level in the DM (**A**) and non-DM (**B**) group.

**Figure S4.** Restricted cubic splines of hsCRP levels in relation to adjusted HR for the risk of MACEs.

| **Table S1.** Relationship between hsCRP and indexes of metabolism by linear regression | | | |
| --- | --- | --- | --- |
| **Variables** | **Adjusted R^2^** | **Coefficient** | **P value** |
| **All patients** |  |  |  |
| HbA1c, % | 0.005 | 0.025 | <0.001 |
| LDL, mmol/L | 0.009 | 0.044 | <0.001 |
| **DM** |  |  |  |
| HbA1c, % | 0.008 | 0.028 | <0.001 |
| LDL, mmol/L | 0.008 | 0.044 | <0.001 |
| **Non-DM** |  |  |  |
| HbA1c, % | 0.006 | 0.054 | <0.001 |
| LDL, mmol/L | 0.010 | 0.045 | <0.001 |

| **Table S2.** Subgroup analysis for the primary endpoint as the unadjusted model | | | | | |
| --- | --- | --- | --- | --- | --- |
| **Variables** | **HsCRP-L/Non-DM** | **HsCRP-H/Non-DM** | **HsCRP-L/ DM** | **HsCRP-H/ DM** | ***P* for interaction** |
| **Age** |  |  |  |  | 0.368 |
| <65 | Reference | 1.52 (1.17-1.97) | 1.40 (1.11-1.75) | 1.86 (1.34-2.58) |  |
| ≥65 | Reference | 0.99 (0.67-1.47) | 1.27 (0.93-1.73) | 1.63 (1.06-2.52) |  |
| **Sex** |  |  |  |  | 0.931 |
| Male | Reference | 1.33 (1.04-1.70) | 1.34 (1.09-1.65) | 1.89 (1.40-2.55) |  |
| Female | Reference | 1.37 (0.86-2.18) | 1.45 (0.98-2.14) | 1.65 (0.97-2.82) |  |
| **BMI** |  |  |  |  | 0.701 |
| <25 | Reference | 1.23 (0.86-1.76) | 1.35 (1.02-1.80) | 2.17 (1.40-3.38) |  |
| ≥25 | Reference | 1.41 (1.08-1.85) | 1.37 (1.08-1.75) | 1.70 (1.23-2.35) |  |
| **Hypertension** |  |  |  |  | 0.506 |
| Yes | Reference | 1.23 (0.94-1.62) | 1.39 (1.12-1.73) | 1.68 (1.24-2.28) |  |
| No | Reference | 1.54 (1.08-2.20) | 1.23 (0.88-1.74) | 2.10 (1.27-3.46) |  |
| **Hyperlipidemia** |  |  |  |  | 0.912 |
| Yes | Reference | 1.41 (1.08-1.84) | 1.41 (1.13-1.75) | 1.88 (1.39-2.55) |  |
| No | Reference | 1.23 (0.85-1.77) | 1.27 (0.90-1.79) | 1.65 (0.98-2.79) |  |
| **Clinical presentation** |  |  |  |  | 0.141 |
| ACS | Reference | 1.18 (0.92-1.52) | 1.49 (1.17-1.91) | 2.15 (1.62-2.85) |  |
| CCS | Reference | 1.49 (1.09-2.04) | 1.22 (0.93-1.61) | 1.58 (1.08-2.31) |  |

| **Table S3.** Subgroup analysis for the primary endpoint as the adjusted model | | | | | |
| --- | --- | --- | --- | --- | --- |
| **Variables** | **HsCRP-L/Non-DM** | **HsCRP-H/Non-DM** | **HsCRP-L/ DM** | **HsCRP-H/ DM** | ***P* for interaction** |
| **Age** |  |  |  |  | 0.449 |
| <65 | Reference | 1.48 (1.14-1.92) | 1.31 (1.04-1.66) | 1.77 (1.27-2.47) |  |
| ≥65 | Reference | 0.97 (0.65-1.44) | 1.25 (0.92-1.70) | 1.52 (0.97-2.37) |  |
| **Sex** |  |  |  |  | 0.950 |
| Male | Reference | 1.30 (1.02-1.66) | 1.26 (1.02-1.55) | 1.73 (1.28-2.34) |  |
| Female | Reference | 1.34 (0.83-2.14) | 1.40 (0.95-2.08) | 1.55 (0.90-2.69) |  |
| **BMI** |  |  |  |  | 0.567 |
| <25 | Reference | 1.18 (0.82-1.69) | 1.25 (0.94-1.68) | 2.04 (1.31-3.19) |  |
| ≥25 | Reference | 1.39 (1.05-1.82) | 1.31 (1.02-1.66) | 1.57 (1.13-2.18) |  |
| **Hypertension** |  |  |  |  | 0.518 |
| Yes | Reference | 1.50 (1.05-2.15) | 1.34 (1.08-1.68) | 1.84 (1.11-3.06) |  |
| No | Reference | 1.22 (0.93-1.60) | 1.15 (0.82-1.63) | 1.64 (1.20-2.23) |  |
| **Hyperlipidemia** |  |  |  |  | 0.894 |
| Yes | Reference | 1.40 (1.07-1.83) | 1.34 (1.08-1.67) | 1.81 (1.33-2.47) |  |
| No | Reference | 1.14 (0.79-1.65) | 1.22 (0.86-1.73) | 1.35 (0.79-2.29) |  |
| **Clinical presentation** |  |  |  |  | 0.204 |
| ACS | Reference | 1.14 (0.89-1.48) | 1.40 (1.10-1.80) | 1.99 (1.50-2.66) |  |
| CCS | Reference | 1.46 (1.07-2.01) | 1.17 (0.89-1.55) | 1.51 (1.02-2.21) |  |

| **Table S4.** Interaction analysis between hsCRP and DM in predicting outcomes | | |
| --- | --- | --- |
| **Outcomes** | **Z value** | **P for interaction value*** |
| MACEs | -0.74 | 0.460 |
| All-cause mortality | -1.22 | 0.223 |
| MI | 0.42 | 0.674 |
| Stroke | -1.88 | 0.060 |
| Unplanned VR | 0.83 | 0.405 |

*P-value is for the test of interaction between diabetes mellitus and hsCRP level (high vs. low) after model adjusted by age, sex, body mass index, current smoking, hypertension, hyperlipidemia, previous stroke, previous PCI, previous coronary artery bypass graft and SYNTAX score.


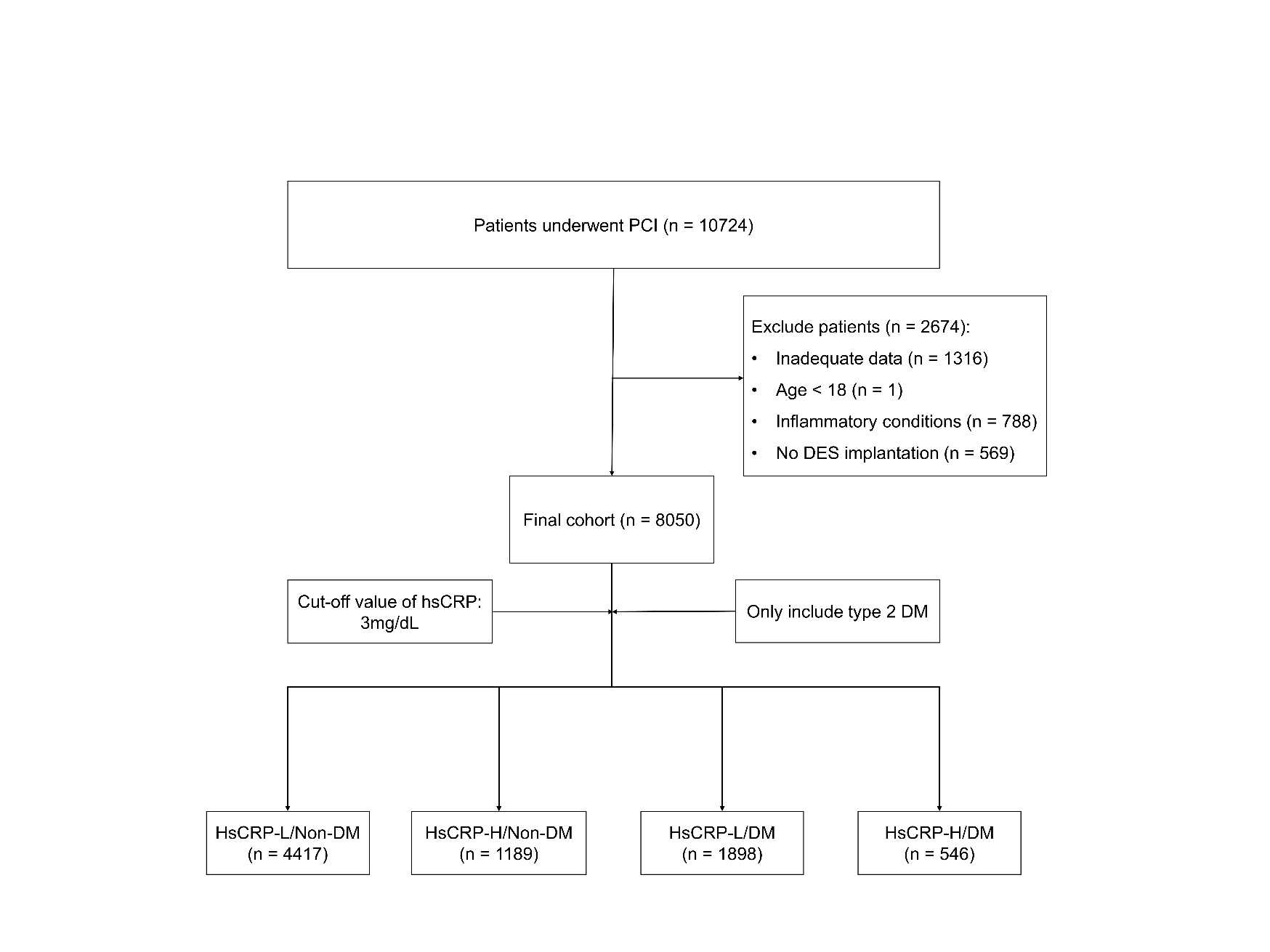


**Figure S1.** Flow chart. PCI, percutaneous coronary intervention; DES, drug-eluting stent; hsCRP, high-sensitivity C-reactive protein; DM, diabetes mellitus.


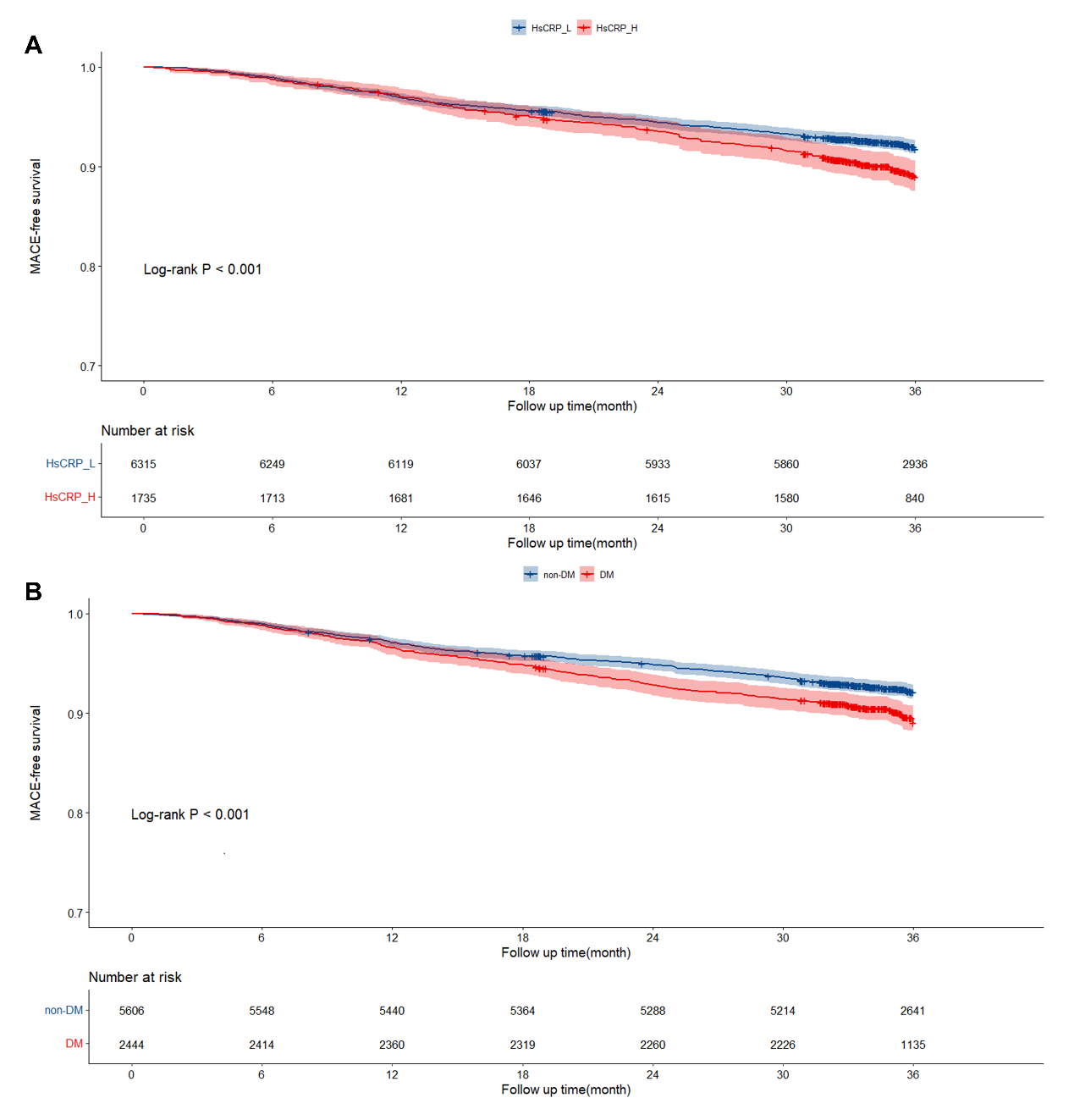


**Figure S2**. Kaplan–Meier analysis for major adverse cardiovascular outcomes according to (**A**) hsCRP and (**B**) diabetes mellitus.


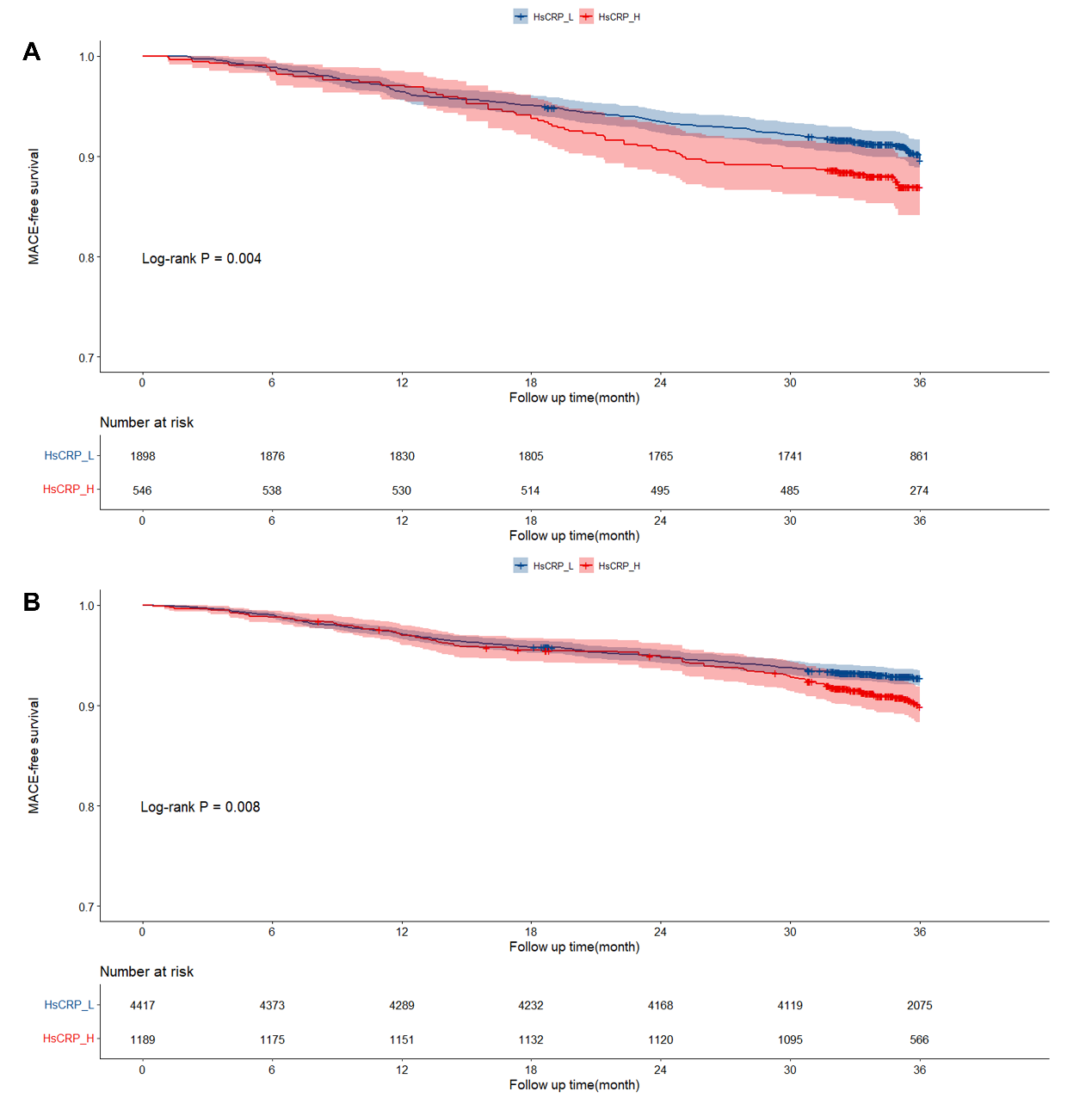


**Figure S3**. Kaplan–Meier analysis for major adverse cardiovascular outcomes according to different hsCRP level in the DM (**A**) and non-DM (**B**) group.


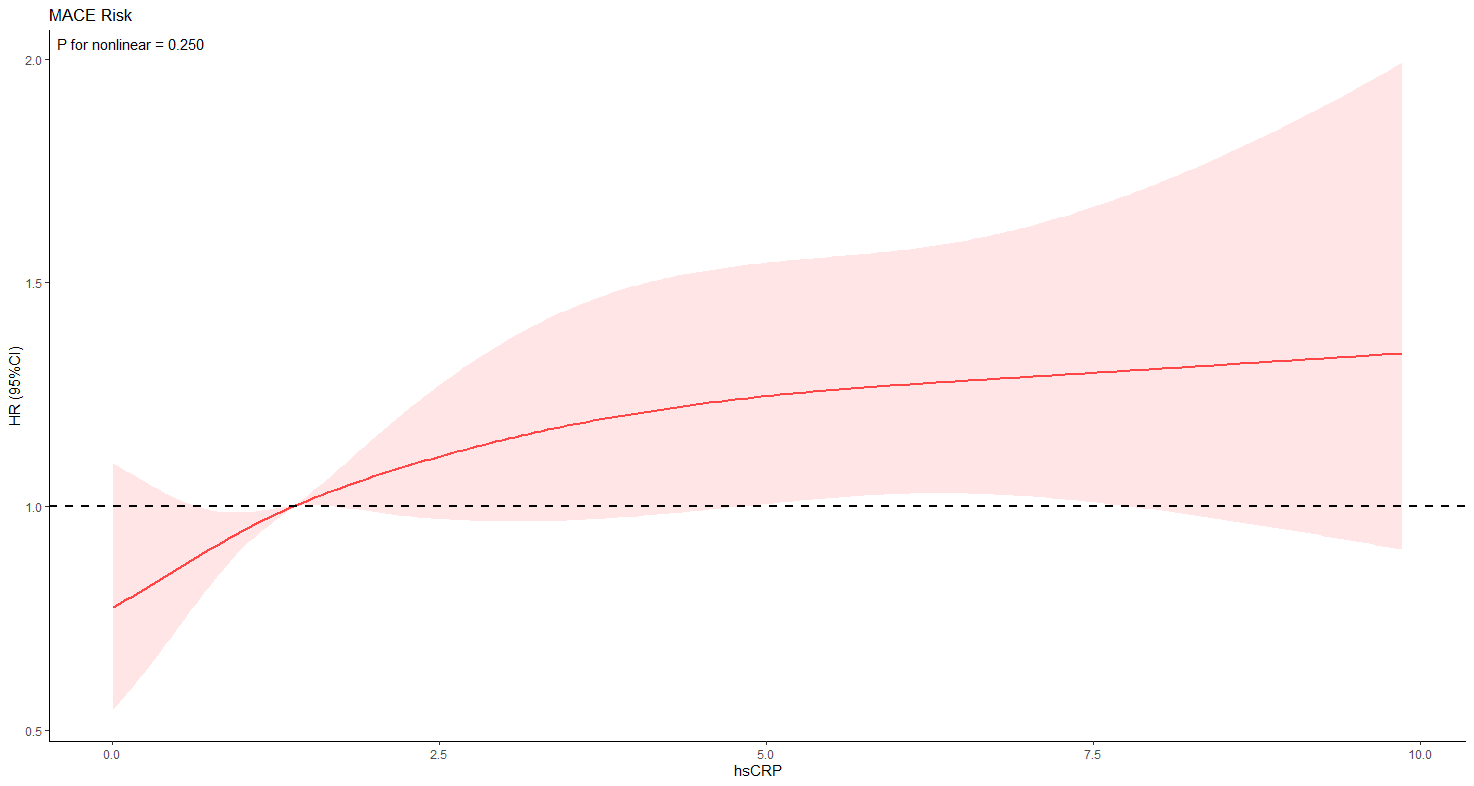


**Figure S4.** Restricted cubic splines of hsCRP levels in relation to adjusted HR for the risk of MACEs.
